# Evaluation of a Transitional Care Program After Hospitalization for Heart Failure in an Integrated Health Care System

**DOI:** 10.1001/jamanetworkopen.2020.27410

**Published:** 2020-12-03

**Authors:** Aileen Baecker, Merry Meyers, Sandra Koyama, Maria Taitano, Heather Watson, Mary Machado, Huong Q. Nguyen

**Affiliations:** 1 Department of Research and Evaluation, Kaiser Permanente Southern California, Pasadena; 2 Regional Offices, Kaiser Permanente Southern California, Pasadena; 3 Baldwin Park Medical Center, Kaiser Permanente Southern California, Pasadena; 4 South Bay Medical Center, Kaiser Permanente Southern California, Pasadena

## Abstract

**Importance:**

Prompted by null findings from several care transition trials and practice changes for heart failure in recent years, leaders from a large integrated health care system aimed to reassess the outcomes of its 10-year multicomponent transitional care program for heart failure (HF-TCP).

**Objective:**

To examine the association of the individual HF-TCP components and their bundle with the primary outcome of all-cause 30-day inpatient or observation stay readmissions.

**Design, Setting, and Participants:**

This retrospective cohort study included patients enrolled in the HF-TCP during an inpatient encounter for heart failure at 13 Kaiser Permanente Southern California hospitals from January 1, 2013, to October 31, 2018, who were followed up from discharge until 30 days, readmission, or death. Data were analyzed from May 7, 2019, to May 1, 2020, with additional review from September 2 to October 1, 2020.

**Exposures:**

Patients received 1 home health visit or telecare (telephone) visit from a registered nurse within 2 days of hospital discharge, a heart failure care manager call within 7 days, and a clinic visit with a physician or a nurse practitioner within 7 days.

**Main Outcomes and Measures:**

Multivariable proportional hazards regression models were used to estimate the probability of 30-day readmission for those who received the individual or bundled HF-TCP components compared with those who did not.

**Results:**

A total of 26 128 patients were included; 57.0% were male, and the mean (SD) age was 73 (13) years. The 30-day readmission rate was 18.1%. Both exposure to a home health visit within 2 days of discharge (hazard ratio [HR], 1.03; 95% CI, 0.96-1.10) and a 7-day heart failure case manager call (HR, 1.08; 95% CI, 0.99-1.18) compared with no visit or call were not associated with a lower rate of readmission. Completion of a 7-day clinic visit was associated with a lower readmission rate (HR, 0.88; 95% CI, 0.81-0.94) compared with no clinic visit. There were no synergistic effects of all 3 components compared with clinic visit alone (HR, 1.05; 95% CI, 0.87-1.28).

**Conclusions and Relevance:**

This study found that HF-TCP as a whole was not associated with a reduction in 30-day readmission rates, although a follow-up clinic visit within 7 days of discharge may be helpful. These findings highlight the importance of continuous quality improvement and refinement of existing clinical programs.

## Key Points

**Question:**

What are the associations between individual components of a real-world transition care program for heart failure (HF-TCP) with all-cause 30-day inpatient or observation stay readmission?

**Findings:**

In a cohort study of 26 128 patients with heart failure, receipt of a home health visit within 2 days of discharge and a 7-day case manager call were not associated with lower rates of readmission. Although completion of a 7-day clinic visit with a physician or a nurse practitioner was marginally associated with lower readmission risk, the HF-TCP as a whole was not associated with lower 30-day readmission rates.

**Meaning:**

These findings suggest that continuous evaluation and refinement of existing clinical programs are critical.

## Teaser

This cohort study examines the association of individual transitional care program components with all-cause 30-day inpatient or observation stay readmission among patients discharged from the hospital after treatment for heart failure.

## Introduction

Heart failure is a common cause of hospitalizations in the Medicare population.^1,2^ Studies focused on improving care transitions and reducing readmission generally use several common intervention elements across 3 domains, including predischarge, postdischarge, and/or bridging interventions.^3,4,5^ Meta-analytic studies concluded that interventions that used a complex and supportive strategy to assess and address contextual issues and limitations in patient capacity were most effective at reducing early readmissions^4^ and that multicomponent interventions that engaged both the patient and family were associated with the highest value for health systems.^3,6^

A meta-analysis of 53 trials of transitional care services with more than 12 000 patients with heart failure^7^ published from 2000 to 2015 found that exposure to home visits by a nurse, disease management clinics, and nurse case management were associated with a 20% to 35% lower risk of all-cause readmissions. Nevertheless, findings from more recent intervention studies tend to have weak to null effects on readmissions.^8^ For instance, a recent large-scale pragmatic study of a multicomponent care transition intervention for 2500 Canadian patients with heart failure across 10 hospitals using a stepped-wedge design (Patient-Centered Care Transitions in Heart Failure [PACT-HF] trial) conducted from 2015 to 2016 found no reductions in all-cause readmission or emergency department visits at 30 days or 3 months compared with usual care.^8^

Prompted by null findings from recent trials of care transition for heart failure as well as substantive changes in standard care transition practices in recent years and suspected implementation drift, clinical and operational leaders from a large integrated health care system, Kaiser Permanente Southern California (KPSC), aimed to reassess the value of its multicomponent transitional care program for heart failure (HF-TCP) nearly 10 years after its initial implementation. The purpose of this analysis was to examine the association of the individual HF-TCP components and the bundled service with the primary outcome of all-cause 30-day inpatient or observation stay readmission.

## Methods

### Design

This study used a retrospective cohort design and was approved by the KPSC institutional review board, which waived the need for informed consent for review of medical records. This report follows the Strengthening the Reporting of Observational Studies in Epidemiology (STROBE) reporting guideline for cohort studies.

### Sample

A total of 28 693 patients enrolled in the HF-TCP program at 13 KPSC medical centers from January 1, 2013, to October 31, 2018. Patients were excluded if they were enrolled in the HF-TCP without an index hospitalization (n = 2565). Only the first index hospitalization for heart failure was included in the analysis. Patients were followed up from discharge until 30 days, readmission, or death.

### Heart Failure Transitional Care Program

The HF-TCP was broadly implemented across KPSC in 2010, modeled after core elements of evidence-based care transition models^9,10,11^ but adapted to the local system context and resources. The overall goal of the HF-TCP was to ensure that patients safely transition from hospital to home by addressing gaps in care, meeting patients’ emerging needs in a timely manner, and empowering patients (with the assistance of their family as appropriate) to engage in self-care. Patients admitted for heart failure who during their hospitalization agreed to enroll in the HF-TCP received a 1-time home health visit (HHV) or telecare (telephone) visit from a registered nurse within 2 days of discharge from the hospital, a call from a heart failure care manager within 7 days, and a physician or nurse practitioner clinic visit in primary or specialty care within 7 days. The HHV could be provided by KPSC registered nurses or contracted to external, non-KPSC home health agencies. The registered nurses received basic training on heart failure management from the clinical program leaders but were not specialists. The HHV typically included medication reconciliation and patient-family education, training, and reinforcement on heart failure self-care behaviors such as fluid intake, sodium restriction, weight monitoring, and implementation of a flexible diuretic plan if appropriate. The telecare calls were conducted by KPSC home health registered nurses and were offered to patients if they declined an HHV. This call included assessing common heart failure symptoms; addressing questions patients might have about their medications, diet, and self-care; and connecting patients to heart failure clinicians as needed. Care manager calls occurred within 7 days of discharge and similarly focused on medication management, self-adjusted diuretic dosing, education on signs and symptoms of an exacerbation, and where to call with concerns. Heart failure care managers were registered nurses, nurse practitioners, or pharmacists. The clinic visits were dedicated postdischarge follow-up visits^12^ with primary care physicians, cardiologists, or cardiology nurse practitioners; these visits focused on medication review and optimization, follow-up on pending test results, and referrals to other medical or social services as needed. Program leaders engaged in ongoing quality, process improvement activities that included audit and feedback of process measures, standard approaches used across other clinical programs.

### Covariates and Outcome

Sociodemographic and clinical characteristics were obtained from the electronic medical record system. The Charlson Comorbidity Index was calculated based on all available diagnostic codes.^13^ Heart failure medications were obtained from pharmacy records in the year before the index hospitalization. Ejection fraction was obtained from echocardiograms closest to the index hospitalization. The laboratory acute physiology score,^14^ a proxy for severity of hospitalization, and LACE (length of stay, acuity of the admission, comorbidity, and emergency department use) readmission risk score^15^ were calculated based on data from the index hospitalization. The primary outcome was all-cause 30-day inpatient or observation stay readmission from clinical and claims records.

### Statistical Analysis

Data were analyzed from May 7, 2019, to May 1, 2020, with additional review performed from September 2 to October 1, 2020. Descriptive statistics were used to describe the study sample by exposure to the HF-TCP components. We used the proportional hazards models of Fine and Gray^16^ to estimate the subdistribution hazard of 30-day all-cause inpatient and observation stay readmissions for those who received the HF-TCP components compared with those who did not at any point during the follow-up period. The 3 HF-TCP components (HHV, care manager call, and clinic visit) were included as independent factors associated with readmission. Death was treated as a competing risk, and the end of the study period was treated as a censoring event. Visits and telephone calls were modeled as time-dependent events to account for immortal time bias^17^; if a patient had more than 1 HHV or telecare visit, care manager call, or clinic visit, then the date of the first contact was used. Variables that were identified as clinically relevant were included in the models. The remainder were optimized using model fit statistics (Akaike information criterion and Schwarz bayesian criterion); variables that did not improve fit and whose inclusion did not appreciably (>10%) affect the estimates were dropped. Covariates in the final model included sociodemographic characteristics (age, sex, race/ethnicity [Black, Hispanic, other, and White]), clinical details (severity of hospitalization [laboratory acute physiology], LACE readmission risk score [<7, 7-10, or ≥11], do-not-resuscitate status [yes or no], functional status at discharge [ambulatory or not], history of myocardial infarction, hypertension, chronic kidney disease [CKD] of stage ≥4, and ejection fraction <40%), medications (vasodilators, β-blockers, and loop diuretics), and other relevant characteristics (received medical financial assistance [yes or no], had any missed appointments [yes or no], enrollment year, and hospital or service area). We also examined the association of the HF-TCP components with outcomes in relevant patient subgroups: ejection fraction (≤40% or >40%), ambulatory vs nonambulatory, CKD stage (<4 or ≥4), and a combination of these groups. A 2-way interaction term between the HF-TCP components and patient subgroup was used to determine whether the association of the HF-TCP components varied based on patient subgroups. All analyses were conducted using SAS, version 9.4 (SAS Institute, Inc). A 2-sided *P* < .05 was considered statistically significant.

## Results

### Sample Characteristics

A total of 26 128 patients with heart failure were eligible for inclusion in the study, of whom 43.0% were female and 57.0% were male, with a mean (SD) age of 73 (13) years (Table 1 and eTable in the Supplement). A total of 4738 encounters (18.1%) were followed by a readmission (inpatient: 3450 [72.8%]; observation stay: 1288 [27.2%]) within 30 days of hospital discharge. A total of 633 deaths occurred within 30 days (2.4%). The mean (SD) number of days from discharge to readmission was 12.2 (8.8).

**Table 1.** Baseline Patient Characteristics by Exposure to HF-TCP Components

Characteristic	HF-TCP component^a^								Total
	2-d HHV/telecare visit				No 2-d HHV/telecare visit				
	CM call plus clinic visit	Clinic visit only	CM call only	None	CM call plus clinic visit	Clinic visit only	CM call only	None	
No. (%) of participants	11 827 (45.3)	1054 (4.0)	3769 (14.4)	625 (2.4)	5337 (20.4)	715 (2.7)	1946 (7.4)	855 (3.3)	26 128 (100)
30-d readmission									
None	10 118 (85.6)	887 (84.2)	2988 (79.3)	351 (56.2)	4582 (85.9)	594 (83.1)	1560 (80.2)	310 (36.3)	21 390 (81.9)
Readmission	1709 (14.4)	167 (15.8)	781 (20.7)	274 (43.8)	755 (14.1)	121 (16.9)	386 (19.8)	545 (63.7)	4738 (18.1)
Inpatient	1227 (10.4)	129 (12.2)	587 (15.6)	197 (31.5)	567 (10.6)	90 (12.6)	298 (15.3)	355 (41.5)	3450 (13.2)
Observation stay	482 (4.1)	38 (3.6)	194 (5.1)	77 (12.3)	188 (3.5)	31 (4.3)	88 (4.5)	190 (22.2)	1288 (4.9)
Time to readmission, mean (SD), d	15.5 (7.7)	13.0 (8.0)	11.7 (8.4)	6.1 (7.1)	15.2 (7.4)	12.7 (8.9)	10.9 (8.3)	2.3 (5.3)	12.2 (8.8)
Death within 30 d	180 (1.5)	29 (2.8)	136 (3.6)	32 (5.1)	101 (1.9)	17 (2.4)	69 (3.5)	69 (8.1)	633 (2.4)
Time to death, mean (SD), d	18.4 (7.3)	17.0 (8.0)	15.3 (8.4)	13.1 (10.0)	19.2 (6.7)	19.2 (7.4)	15.4 (8.5)	7.2 (8.9)	16.0 (8.7)
Year^b^									
2013	2106 (37.5)	189 (3.4)	1091 (19.4)	189 (3.4)	1021 (18.2)	216 (3.8)	542 (9.7)	261 (4.6)	5615 (21.5)
2014	1854 (42.5)	105 (2.4)	642 (14.7)	75 (1.7)	1020 (23.4)	91 (2.1)	424 (9.7)	147 (3.4)	4358 (16.7)
2015	1299 (31.1)	97 (2.3)	365 (8.7)	47 (1.1)	1540 (36.8)	181 (4.3)	476 (11.4)	176 (4.2)	4181 (16.0)
2016	2325 (55.3)	153 (3.6)	600 (14.3)	80 (1.9)	697 (16.6)	56 (1.3)	202 (4.8)	88 (2.1)	4201 (16.1)
2017	2117 (51.9)	383 (9.4)	557 (13.7)	175 (4.3)	469 (11.5)	116 (2.8)	151 (3.7)	108 (2.6)	4076 (15.6)
2018	2126 (57.5)	127 (3.4)	514 (13.9)	59 (1.6)	590 (16.0)	55 (1.5)	151 (4.1)	75 (2.0)	3697 (14.1)
Sociodemographic characteristic									
Age, mean (SD), y	73.5 (13.2)	74.7 (13.1)	73.4 (13.8)	74.1 (12.7)	72.1 (13.6)	71.4 (12.9)	70.8 (14.4)	71.5 (14.2)	72.9 (13.5)
Female	5141 (43.5)	438 (41.6)	1720 (45.6)	306 (49.0)	2176 (40.8)	301 (42.1)	818 (42.0)	348 (40.7)	11 248 (43.0)
Race/ethnicity									
Black	1678 (14.2)	134 (12.7)	635 (16.8)	106 (17.0)	843 (15.8)	129 (18.0)	395 (20.3)	121 (14.2)	4041 (15.5)
Hispanic	3181 (26.9)	276 (26.2)	874 (23.2)	148 (23.7)	1395 (26.1)	176 (24.6)	444 (22.8)	202 (23.6)	6696 (25.6)
Other	1084 (9.2)	82 (7.8)	314 (8.3)	49 (7.8)	461 (8.6)	43 (6.0)	156 (8.0)	75 (8.8)	2264 (8.7)
White	5884 (49.8)	562 (53.3)	1946 (51.6)	322 (51.5)	2638 (49.4)	367 (51.3)	951 (48.9)	457 (53.5)	13 127 (50.2)
Married or partnered	6287 (53.2)	585 (55.5)	1775 (47.1)	299 (47.8)	2870 (53.8)	388 (54.3)	906 (46.6)	413 (48.3)	13 523 (51.8)
Insurance status									
Commercial or private	2929 (24.8)	230 (21.8)	940 (24.9)	131 (21.0)	1534 (28.7)	189 (26.4)	593 (30.5)	242 (28.3)	6788 (26.0)
Medicaid or dual	786 (6.6)	61 (5.8)	222 (5.9)	35 (5.6)	319 (6.0)	40 (5.6)	122 (6.3)	51 (6.0)	1636 (6.3)
Medicare	8055 (68.1)	757 (71.8)	2569 (68.2)	453 (72.5)	3448 (64.6)	478 (66.9)	1208 (62.1)	548 (64.1)	17 516 (67.0)
Other	57 (0.5)	6 (0.6)	38 (1.0)	6 (1.0)	36 (0.7)	8 (1.1)	23 (1.2)	14 (1.6)	188 (0.7)
Received medical financial assistance^c^	1017 (8.6)	98 (9.3)	357 (9.5)	68 (10.9)	506 (9.5)	75 (10.5)	205 (10.5)	89 (10.4)	2415 (9.2)
Missed appointments^c^	8212 (69.4)	734 (69.6)	2687 (71.3)	494 (79.0)	3734 (70.0)	519 (72.6)	1402 (72.0)	635 (74.3)	18 417 (70.5)
Prehospitalization clinical characteristics									
Charlson Comorbidity Index, mean (SD)	6.5 (3.1)	6.7 (3.3)	6.4 (3.3)	6.9 (3.4)	6.4 (3.2)	6.7 (3.3)	6.0 (3.3)	6.4 (3.3)	6.4 (3.2)
Cancer	2631 (22.2)	241 (22.9)	830 (22.0)	147 (23.5)	1175 (22.0)	158 (22.1)	370 (19.0)	172 (20.1)	5724 (21.9)
Cerebrovascular disease	3538 (29.9)	343 (32.5)	1172 (31.1)	205 (32.8)	1569 (29.4)	234 (32.7)	560 (28.8)	278 (32.5)	7899 (30.2)
Chronic pulmonary disease	7328 (62.0)	652 (61.9)	2287 (60.7)	388 (62.1)	3344 (62.7)	458 (64.1)	1162 (59.7)	521 (60.9)	16 140 (61.8)
Dementia	740 (6.3)	69 (6.5)	282 (7.5)	57 (9.1)	241 (4.5)	40 (5.6)	112 (5.8)	54 (6.3)	1595 (6.1)
Diabetes with complications	5262 (44.5)	465 (44.1)	1543 (40.9)	284 (45.4)	2351 (44.1)	328 (45.9)	709 (36.4)	365 (42.7)	11 307 (43.3)
Myocardial infarction	4694 (39.7)	438 (41.6)	1510 (40.1)	297 (47.5)	2215 (41.5)	316 (44.2)	801 (41.2)	389 (45.5)	10 660 (40.8)
Renal disease	6861 (58.0)	626 (59.4)	2152 (57.1)	375 (60.0)	3118 (58.4)	419 (58.6)	1034 (53.1)	481 (56.3)	15 066 (57.7)
CKD stage ≥4	1491 (12.6)	134 (12.7)	433 (11.5)	81 (13.0)	668 (12.5)	78 (10.9)	207 (10.6)	101 (11.8)	3193 (12.2)
Ejection fraction, mean (SD), %	44.9 (15.9)	45.0 (15.5)	44.7 (16.1)	44.6 (16.4)	43.4 (16.1)	44.6 (15.1)	42.4 (16.2)	43.2 (16.3)	44.3 (16.0)
Ejection fraction <40%	4373 (37.0)	372 (35.3)	1439 (38.2)	224 (35.8)	2183 (40.9)	252 (35.2)	857 (44.0)	347 (40.6)	10 047 (38.5)
Medication used									
Vasodilator^d^	2740 (62.7)	227 (61.0)	945 (65.7)	136 (60.7)	1381 (63.3)	171 (67.9)	533 (62.2)	249 (71.8)	6382 (63.5)
β-blocker^e^	2681 (61.3)	227 (61.0)	946 (65.7)	137 (61.2)	1371 (62.8)	166 (65.9)	549 (64.1)	235 (67.7)	6312 (62.8)
Diuretic^f^	6248 (52.8)	574 (54.5)	2077 (55.1)	345 (55.2)	2880 (54.0)	377 (52.7)	1042 (53.5)	456 (53.3)	13 999 (53.6)
Heart failure index hospitalization									
LACE readmission score, mean (SD)^g^	11.0 (2.6)	11.1 (2.6)	10.6 (2.8)	11.1 (2.7)	10.8 (2.6)	10.9 (2.8)	10.2 (2.9)	10.3 (3.0)	10.8 (2.7)
<7	693 (5.9)	53 (5.0)	330 (8.8)	32 (5.1)	357 (6.7)	52 (7.3)	229 (11.8)	86 (10.1)	1832 (7.0)
7-10	4065 (34.4)	370 (35.1)	1615 (42.8)	248 (39.7)	1971 (36.9)	262 (36.6)	857 (44.0)	373 (43.6)	9761 (37.4)
≥11	7069 (59.8)	631 (59.9)	1824 (48.4)	345 (55.2)	3009 (56.4)	401 (56.1)	860 (44.2)	396 (46.3)	14 535 (55.6)
Laboratory acute physiology score, mean (SD)^h^	85.9 (28.7)	84.8 (28.6)	86.5 (30.5)	87.1 (30.0)	84.8 (28.6)	83.2 (28.6)	83.0 (29.3)	81.9 (36.8)	85.3 (29.4)
Length of stay, mean (SD), d	4.2 (4.4)	4.3 (3.9)	3.9 (5.5)	4.1 (3.9)	3.7 (3.4)	4.5 (5.4)	3.5 (3.7)	3.3 (4.5)	4.0 (4.3)
Do not resuscitate code status	1985 (16.8)	191 (18.1)	757 (20.1)	124 (19.8)	820 (15.4)	71 (9.9)	326 (16.8)	151 (17.7)	4425 (16.9)
Functional status^i^									
Nonambulatory	1358 (11.5)	128 (12.1)	635 (16.8)	127 (20.3)	642 (12.0)	118 (16.5)	300 (15.4)	140 (16.4)	3448 (13.2)
Ambulates with assistance	6796 (57.5)	579 (54.9)	2002 (53.1)	313 (50.1)	2948 (55.2)	333 (46.6)	1019 (52.4)	434 (50.8)	14 424 (55.2)
Ambulates independently	3455 (29.2)	333 (31.6)	1016 (27.0)	172 (27.5)	1628 (30.5)	244 (34.1)	568 (29.2)	245 (28.7)	7661 (29.3)

Abbreviations: CKD, chronic kidney disease; CM, care manager; HF-TCP, heart failure transitional care program; HHV, home health visit; LACE, length of stay, acuity of the admission, comorbidity, and emergency department use.

^a^ Patients were grouped between those who received vs did not receive the 2-day HHV or telecare call from a registered nurse. These groups were divided by the other components of the HF-TCP received (CM call and clinic visit with a physician or nurse practitioner within 7 days of discharge). Unless otherwise indicated, data are expressed as number (percentage) of patients based on column totals. Percentages have been rounded and may not total 100.

^b^ Percentages are calculated from row totals.

^c^ Collected 12 months before index hospitalization.

^d^ Includes angiotensin-converting enzyme inhibitor, angiotensin II receptor blocker, angiotensin receptor neprilysin inhibitor, or hydralazine-nitrate 12 months before the index hospitalization for patients with ejection fraction of less than 40%.

^e^ Includes metoprolol, carvedilol, or bisoprolol 12 months before the index hospitalization for patients with ejection fraction of less than 40%.

^f^ Includes furosemide or bumetanide 12 months before the index hospitalization for all patients.

^g^ Higher scores indicate increased risk of readmission.

^h^ Scores range from 0 to 320, with higher scores indicating greater severity of hospitalization.

^i^ Within 24 hours of hospital discharge. Percentages may not total 100 owing to missing data.

### HF-TCP Components and 30-Day All-Cause Inpatient and Observation Stay Readmission

#### HHV and Telecare Call Within 2 Days of Discharge

Two-thirds of the encounters (66.1%) were followed by an HHV (KPSC, 8282 [31.7%]; non-KPSC, 5255 [20.1%]) or telecare call (5214 [20.0%]) within 2 days of discharge. Readmissions were less likely among patients who had an KPSC HHV (1436 [17.3%]), non-KPSC HHV (926 [17.6%]), or a telecare call (793 [15.2%]) compared with those without any contact from the HF-TCP within 2 days of discharge (1807 [20.4%]). Over time, KPSC HHVs became less common (2251 [45.4%] of encounters in 2013 vs 762 [20.6%] in 2018), whereas non-KPSC HHVs (797 [14.2%] vs 1282 [34.7%]) and telecare calls (290 [5.2%] vs 1125 [30.4%]) increased. In adjusted models, exposure to an HHV or telecare call within 2 days of discharge was not associated with a lower rate of readmission (hazard ratio [HR], 1.03; 95% CI, 0.96-1.10) compared with no contact. Home health visits by KPSC registered nurses were not different from non-KPSC HHVs (HR, 0.95; 95% CI, 0.86-1.04) or telecare calls (HR, 1.00; 95% CI, 0.87-1.15) (Table 2).

**Table 2.** Exposures to HF-TCP Components and 30-Day All-Cause Inpatient and Observation Stay Readmission Risk (N = 26 128)

Component^a^	HR (95% CI)^b^
2-d HHV or telecare vs none	1.03 (0.96-1.10)
KP HHV vs no HHV visit	1.01 (0.94-1.09)
Non-KP HHV vs no HHV	1.07 (0.98-1.17)
KP HHV vs non-KP HHV	0.95 (0.86-1.04)
2-d Telecare	
KP HHV vs telecare	1.00 (0.87-1.15)
7-d CM call vs no CM call	1.08 (0.99-1.18)
HHV/telecare plus CM vs HHV/telecare	1.16 (1.00-1.34)
7-d Clinic visit vs no clinic visit	0.88 (0.81-0.94)
HHV/telecare or CM call plus clinic visit vs clinic visit only	1.03 (0.85-1.25)
HHV/telecare plus CM call plus clinic visit vs clinic visit only	1.05 (0.87-1.28)

Abbreviations: CM, care manager; HF-TCP, heart failure transitional care program; HHV, home health visit; HR, hazard ratio; KP, Kaiser Permanente.

^a^ The HHV or telecare call with a registered nurse occurred within 2 days of hospital discharge; CM call and clinic visit with a physician or a nurse practitioner occurred within 7 days of hospital discharge.

^b^ Proportional hazards models of Fine and Gray accounted for sociodemographics (age, sex, race/ethnicity), clinical (severity of hospitalization [laboratory acute physiology], LACE [length of stay, acuity of the admission, comorbidity, and emergency department use], readmission risk score [<7, 7-10, or ≥11], do-not-resuscitate status [yes or no], functional status at discharge [ambulatory or not], history of myocardial infarction, hypertension, chronic kidney disease stage ≥4, and ejection fraction <40%), medications (vasodilators, β-blockers, and loop diuretics), and other relevant characteristics (received medical financial assistance [yes or no], had any missed appointments [yes or no], enrollment year, and hospital/service area). All covariates were significantly associated with readmission except use of vasodilators and ejection fraction of less than 40%.

#### Heart Failure Care Manager Call Within 7 Days of Discharge

Most patients received a care manager call (87.6% of encounters), although receipt of the call was not associated with lower readmission in adjusted models (HR, 1.08; 95% CI, 0.99-1.18). When the care manager call was coupled with the HHV or telecare call, this bundle was marginally associated with higher readmission compared to the HHV or telecare call alone (HR, 1.16; 95% CI, 1.00-1.34) (Table 2).

#### Clinic Visit Within 7 Days of Discharge

Most patients completed a visit with a physician or nurse practitioner (72.5%). In adjusted models, this component was associated with a lower risk of readmission (HR, 0.88; 95% CI, 0.81-0.94) compared with no clinic visit within 7 days. The combination of a clinic visit plus HHV or telecare call was not associated with lower readmission compared to a clinic visit alone (HR, 1.03; 95% CI, 0.85-1.25). Similarly, there were no synergistic effects of all 3 bundled components compared with the clinic visit alone (HR, 1.05; 95% CI, 0.87-1.28).

### HF-TCP Components and 30-Day All-Cause Inpatient and Observation Stay Readmission

The HHV and telecare component appeared to be marginally associated with lower readmission for patients with ejection fraction of 40% or less (HR, 0.91; 95% CI, 0.82-1.00) vs an ejection fraction of greater than 40% (HR, 1.11; 95% CI, 1.02-1.21) (*P* = .002 for interaction), for patients who had a combination of an ejection fraction of 40% or less and CKD stage of less than 4 (HR, 0.91; 95% CI, 0.81-1.01) compared with patients who did not meet this criteria (HR, 1.09; 95% CI, 1.01-1.19; *P* = .004), and for patients who had a combination of these ejection fraction and CKD criteria and were ambulatory (HR, 0.89; 95% CI, 0.79-1.00) compared with those who did not meet these criteria (HR, 1.09; 95% CI, 1.00-1.17; *P* = .004). There were no other meaningful interactions across these clinical subgroups on the care manager calls, clinic visits, or all 3 components combined (Table 3).

**Table 3.** Response of Subgroups to HF-TCP Components and 30-Day All-Cause Inpatient and Observation Stay Readmission Risk^a^

Patient subgroup	No. of patients	2-d HHV or telecare		7-d CM call		7-d Clinic visit		HHV or telecare plus CM call plus clinic visit	
		HR (95% CI)	*P* value^b^	HR (95% CI)	*P* value^b^	HR (95% CI)	*P* value^b^	HR (95% CI)	*P* value^b^
All	26 081	1.03 (0.96-1.10)	NA	1.08 (0.99-1.18)	NA	0.88 (0.81-0.94)	NA	0.95 (0.89-1.02)	NA
Ejection fraction, %									
>40	16 054	1.11 (1.02-1.21)	.002	1.11 (1.00-1.23)	.35	0.87 (0.80-0.95)	.96	0.97 (0.89-1.05)	.38
≤40	10 027	0.91 (0.82-1.00)		1.03 (0.91-1.18)		0.88 (0.79-0.98)		0.92 (0.82-1.02)	
CKD stage									
<4 or Missing	22 892	1.02 (0.95-1.10)	.65	1.05 (0.96-1.16)	.08	0.85 (0.79-0.92)	.05	0.94 (0.87-1.01)	.26
≥4	3189	1.06 (0.91-1.24)		1.25 (1.03-1.52)		1.00 (0.86-1.17)		1.03 (0.88-1.20)	
Functional status at discharge									
Ambulatory	22 052	1.02 (0.95-1.10)	.87	1.08 (0.98-1.19)	.58	0.88 (0.81-0.95)	.90	0.96 (0.90-1.04)	.50
Nonambulatory	3443	1.03 (0.89-1.20)		1.03 (0.87-1.22)		0.87 (0.75-1.00)		0.91 (0.78-1.06)	
Ejection fraction ≤40% plus CKD stage <4									
Yes	9139	0.91 (0.81-1.01)	.004	0.97 (0.85-1.11)	.04	0.84 (0.75-0.93)	.29	0.89 (0.80-1.00)	.16
No	16 942	1.09 (1.01-1.19)		1.13 (1.02-1.26)		0.90 (0.82-0.97)		0.98 (0.90-1.06)	
Ejection fraction ≤40% plus CKD stage <4 plus ambulatory									
Yes	7932	0.89 (0.79-1.00)	.004	0.97 (0.84-1.12)	.06	0.82 (0.73-0.92)	.17	0.90 (0.80-1.02)	.29
No	18 149	1.09 (1.00-1.17)		1.12 (1.02-1.24)		0.90 (0.83-0.97)		0.97 (0.90-1.05)	

Abbreviations: CKD, chronic kidney disease; CM, care manager; HF-TCP, heart failure transitional care program; HHV, home health visit; HR, hazard ratio; NA, not applicable.

^a^ The HHV or telecare call with a registered nurse occurred within 2 days of hospital discharge; CM call and clinic visit with a physician or a nurse practitioner occurred within 7 days of hospital discharge.

^b^ Indicates *P* value for interaction.

## Discussion

This evaluation of a real-world multicomponent transitional care program for patients hospitalized for heart failure showed that the HF-TCP as a whole was not associated with a reduction in all-cause 30-day inpatient or observation stay readmission, although one of the program components, a follow-up clinic visit within 7 days of discharge, may be helpful; the HHV with a registered nurse and a heart failure care manager telephone call conducted within 2 and 7 days of discharge, respectively, were not associated with lower readmission. These results, although disappointing, were not surprising given findings from recent studies.^8,18^ Nonetheless, an important consideration is the relatively low 30-day mortality (2.4%) and readmission rates (18.1%) in this study sample compared with a 7.6% mortality rate from the Get With the Guidelines-Heart Failure Registry^18^ and the national heart failure 30-day readmission rate of 21.5% from the Centers for Medicare & Medicaid Services, which leaves little room for further improvements.

The association between completing a follow-up visit and lower readmission is consistent with an earlier study in a large cohort of patients on the medicine service,^12^ as well as other studies of patients with heart failure specifically,^19,20^ and aligns with current reimbursement policies for transitional care management, although those who are well enough to complete a clinic visit may be less likely to be readmitted to the hospital. The rate of 7-day clinic follow-up in our study (72.5%) is rather impressive compared with other published studies reporting rates of postdischarge follow-up visits of approximately one-third.^21,22^ This may, however, reflect the fact that patients who agree to participate in the HF-TCP are likely to be more adherent to care recommendations compared with the general population with heart failure. Greater use of telehealth going forward could facilitate more robust and seamless handoffs to outpatient care, especially for vulnerable, older patients with functional limitations and/or transportation challenges.^22^

The challenge with evaluating the effects of the heart failure care manager calls was that 87.6% of patients received at least 1 call, leaving a very small control group that was not exposed to a call for comparison. Given that postdischarge calls are now an expected standard practice for medium- to high-risk patients that is reimbursed by private and public payers, future research is best directed at ensuring high-quality, effective interactions during these brief calls to maximize value for patients, families, health systems, and payers.

The lack of an association from the 1-time HHV with a nurse was disappointing but not surprising, because program leaders had been concerned about the quality of the interactions between nonspecialist home health nurses and patients and families for education and training on medications and self-care behaviors. It can be extremely challenging to provide ongoing training and maintain quality assurance with a large home care workforce that typically experiences high turnover and chronic staffing shortages. Our experience with implementing nursing HHVs at scale is very different from that of previous, relatively small-scale efficacy studies of nurse HHVs. For instance, a meta-analysis of 53 trials of transitional care services with more than 120 000 patients with heart failure reported that nurse HHVs were associated with a 35% reduction in all-cause readmission. These studies often included multiple visits by highly trained heart failure nurses,^7^ which is simply not scalable nor sustainable for systems that are responsible for managing a large population of patients with heart failure.

It is important to note that although the home health nurses conducted these visits, the visits were not considered standard, Medicare-certified home visits with the regulatory requirements for patients to be homebound and having a skilled need and did not come with the extensive documentation. The fact that a single HHV was provided in this clinical program as opposed to the multiple visits in more ideal grant-funded research studies^7^ may have contributed to the null effects. Moreover, the home health nurses were not as well integrated into the heart failure care management team as the specialized nurses in published studies.^7^ Greater efforts to improve handoff communications and coordination across the inpatient, home health, and outpatient care teams may improve outcomes, as well as consideration for instituting additional home visits for higher-risk patients.

However, our subgroup analyses do offer insights into how the 1-time nurse HHV could be refined to target a subset of patients further upstream who might have less severe heart failure, fewer comorbidities, and nonsevere functional impairment, wherein a brief home-based interaction might have positive effects, but this targeted approach will need to be tested. Remote biometric monitoring may offer some promise for transitional care from a scaling perspective; however, published trials^23,24,25^ have had mixed results, and more rigorous investigation is required to determine which patient subgroups would benefit most from ongoing surveillance.

Finally, despite the high mortality rate 12 months after hospitalization for patients with heart failure^26^ and recommendations from professional societies on the need to integrate palliative care with heart failure care,^27^ very limited data exist regarding the effectiveness of primary or secondary palliative care services for heart failure.^28^ Only a few small studies with mixed findings regarding the effect on proximal patient-centered outcomes such as quality of life are available. None of the heart failure palliative care studies^29,30,31^ thus far have been powered to detect reductions in use of acute care services. Interestingly, the PACT-HF study,^8^ which was not framed as a palliative care intervention but had many palliative care elements, reported significant improvements in measures such as perception of discharge preparedness, quality of care transition, and quality of life, although there were no reductions in use of services.

### Limitations

There are several notable limitations with this study. The analytical cohort represents only patients who agreed to participate in the HF-TCP. Although this limits the generalizability of the findings to the broad patient population with heart failure that was discharged from Kaiser Permanente hospitals to home, the findings likely represent the best-case scenario in terms of intervention effects for patients who were receptive to the program. We were unable to measure the content and heterogeneity in the implementation quality for each program component within and across the 13 sites and could only rely on whether an encounter occurred; the overstretched checklist home care culture may have prioritized task completions over quality, and thus the null findings for the HHV in particular could reflect a failure in implementation rather than a lack of effectiveness. The HF-TCP did not routinely capture other patient-centered outcomes (eg, quality of life, discharge preparedness), and thus we do not know whether the program had a positive effect on these important, proximal measures.^8^ The follow-up duration of 30 days was short, although the HF-TCP was designed to be a time-limited intervention. We did not correct for multiple comparisons that could have generated significant findings by chance alone. Finally, omission of unmeasured confounders, such as exposure to other care transition interventions, adherence to heart failure treatment, and socioeconomic risks, may have also biased some of the findings.

## Conclusions

We found that a multicomponent transitional care program for patients with heart failure, as implemented in the real world, was not associated with a reduction in 30-day all-cause inpatient or observation stay readmission, although 1 program component, a follow-up clinic visit with a physician or nurse practitioner within 7 days of discharge, may be helpful; the nurse HHV and a heart failure care manager telephone call conducted within 2 and 7 days of discharge, respectively, were not associated with lower readmission rates. These findings highlight the importance of ongoing evaluation, continuous improvement, and refinement of existing clinical programs for any learning health care system to maximize the value of its services, especially for a challenging clinical condition such as heart failure.

## Notes

Supplement. **eTable.** Characteristics of HF-TCP Patients Who Were Readmitted vs not Readmitted

## References

[1] Wadhera RK, Joynt Maddox KE, Kazi DS, Shen C, Yeh RW Hospital revisits within 30 days after discharge for medical conditions targeted by the Hospital Readmissions Reduction Program in the United States: national retrospective analysis. BMJ. 2019;366:l4563. doi:10.1136/bmj.l4563 31405902 PMC6689820

[2] Parikh KS, Sheng S, Hammill BG, Characteristics of acute heart failure hospitalizations based on presenting severity. Circ Heart Fail. 2019;12(1):e005171. doi:10.1161/CIRCHEARTFAILURE.118.005171 30630340

[3] Nuckols TK, Keeler E, Morton S, Economic evaluation of quality improvement interventions designed to prevent hospital readmission: a systematic review and meta-analysis. JAMA Intern Med. 2017;177(7):975-985. doi:10.1001/jamainternmed.2017.1136 28558095 PMC5710454

[4] Leppin AL, Gionfriddo MR, Kessler M, Preventing 30-day hospital readmissions: a systematic review and meta-analysis of randomized trials. JAMA Intern Med. 2014;174(7):1095-1107. doi:10.1001/jamainternmed.2014.1608 24820131 PMC4249925

[5] Hansen LO, Young RS, Hinami K, Leung A, Williams MV Interventions to reduce 30-day rehospitalization: a systematic review. Ann Intern Med. 2011;155(8):520-528. doi:10.7326/0003-4819-155-8-201110180-00008 22007045

[6] Rodakowski J, Rocco PB, Ortiz M, Caregiver integration during discharge planning for older adults to reduce resource use: a metaanalysis. J Am Geriatr Soc. 2017;65(8):1748-1755. doi:10.1111/jgs.14873 28369687 PMC5555776

[7] Van Spall HGC, Rahman T, Mytton O, Comparative effectiveness of transitional care services in patients discharged from the hospital with heart failure: a systematic review and network meta-analysis. Eur J Heart Fail. 2017;19(11):1427-1443. doi:10.1002/ejhf.765 28233442

[8] Van Spall HGC, Lee SF, Xie F, Effect of patient-centered transitional care services on clinical outcomes in patients hospitalized for heart failure: the PACT-HF randomized clinical trial. JAMA. 2019;321(8):753-761. doi:10.1001/jama.2019.0710 30806695 PMC6439867

[9] Naylor MD, Brooten DA, Campbell RL, Maislin G, McCauley KM, Schwartz JS Transitional care of older adults hospitalized with heart failure: a randomized, controlled trial. J Am Geriatr Soc. 2004;52(5):675-684. doi:10.1111/j.1532-5415.2004.52202.x 15086645

[10] Naylor MD, Brooten D, Campbell R, Comprehensive discharge planning and home follow-up of hospitalized elders: a randomized clinical trial. JAMA. 1999;281(7):613-620. doi:10.1001/jama.281.7.613 10029122

[11] Coleman EA, Parry C, Chalmers S, Min SJ The care transitions intervention: results of a randomized controlled trial. Arch Intern Med. 2006;166(17):1822-1828. doi:10.1001/archinte.166.17.1822 17000937

[12] Shen E, Koyama SY, Huynh DN, Association of a dedicated post-hospital discharge follow-up visit and 30-day readmission risk in a Medicare Advantage population. JAMA Intern Med. 2017;177(1):132-135. doi:10.1001/jamainternmed.2016.7061 27893040

[13] Charlson ME, Pompei P, Ales KL, MacKenzie CR A new method of classifying prognostic comorbidity in longitudinal studies: development and validation. J Chronic Dis. 1987;40(5):373-383. doi:10.1016/0021-9681(87)90171-8 3558716

[14] Escobar GJ, Gardner MN, Greene JD, Draper D, Kipnis P Risk-adjusting hospital mortality using a comprehensive electronic record in an integrated health care delivery system. Med Care. 2013;51(5):446-453. doi:10.1097/MLR.0b013e3182881c8e 23579354

[15] van Walraven C, Dhalla IA, Bell C, Derivation and validation of an index to predict early death or unplanned readmission after discharge from hospital to the community. CMAJ. 2010;182(6):551-557. doi:10.1503/cmaj.091117 20194559 PMC2845681

[16] Fine JP, Gray RJ A proportional hazards model for the subdistribution of a competing risk. J Am Stat Assoc. 1999;94:496-509. doi:10.1080/01621459.1999.10474144

[17] Zhou Z, Rahme E, Abrahamowicz M, Pilote L Survival bias associated with time-to-treatment initiation in drug effectiveness evaluation: a comparison of methods. Am J Epidemiol. 2005;162(10):1016-1023. doi:10.1093/aje/kwi307 16192344

[18] Bergethon KE, Ju C, DeVore AD, Trends in 30-day readmission rates for patients hospitalized with heart failure: findings from the Get With the Guidelines-Heart Failure Registry. Circ Heart Fail. 2016;9(6):e002594. doi:10.1161/CIRCHEARTFAILURE.115.002594 27301467 PMC4928632

[19] Health Quality Ontario Effect of early follow-up after hospital discharge on outcomes in patients with heart failure or chronic obstructive pulmonary disease: a systematic review. Ont Health Technol Assess Ser. 2017;17(8):1-37. 28638496 PMC5466361

[20] Edmonston DL, Wu J, Matsouaka RA, Association of post-discharge specialty outpatient visits with readmissions and mortality in high-risk heart failure patients. Am Heart J. 2019;212:101-112. doi:10.1016/j.ahj.2019.03.005 30978555

[21] Baker H, Oliver-McNeil S, Deng L, Hummel SL Regional hospital collaboration and outcomes in Medicare heart failure patients: see you in 7. JACC Heart Fail. 2015;3(10):765-773. doi:10.1016/j.jchf.2015.06.007 26364256 PMC4701212

[22] DeVore AD, Cox M, Eapen ZJ, Temporal trends and variation in early scheduled follow-up after a hospitalization for heart failure: findings from Get With The Guidelines–Heart Failure. Circ Heart Fail. 2016;9(1):e002344. doi:10.1161/CIRCHEARTFAILURE.115.002344 26754624

[23] Ong MK, Romano PS, Edgington S, ; Better Effectiveness After Transition–Heart Failure (BEAT-HF) Research Group Effectiveness of remote patient monitoring after discharge of hospitalized patients with heart failure: the Better Effectiveness After Transition–Heart Failure (BEAT-HF) randomized clinical trial. JAMA Intern Med. 2016;176(3):310-318. doi:10.1001/jamainternmed.2015.7712 26857383 PMC4827701

[24] Gonzalez Garcia M, Fatehi F, Bashi N, A review of randomized controlled trials utilizing telemedicine for improving heart failure readmission: can a realist approach bridge the translational divide? Clin Med Insights Cardiol. 2019;13:1179546819861396. doi:10.1177/1179546819861396 31316270 PMC6620724

[25] Bekelman DB, Plomondon ME, Carey EP, Primary results of the Patient-Centered Disease Management (PCDM) for Heart Failure Study: a randomized clinical trial. JAMA Intern Med. 2015;175(5):725-732. doi:10.1001/jamainternmed.2015.0315 25822284

[26] Tromp J, Bamadhaj S, Cleland JGF, Post-discharge prognosis of patients admitted to hospital for heart failure by world region, and national level of income and income disparity (REPORT-HF): a cohort study. Lancet Glob Health. 2020;8(3):e411-e422. doi:10.1016/S2214-109X(20)30004-8 32087174

[27] Hunt SA, Abraham WT, Chin MH, ; American College of Cardiology; American Heart Association Task Force on Practice Guidelines; American College of Chest Physicians; International Society for Heart and Lung Transplantation; Heart Rhythm Society ACC/AHA 2005 Guideline Update for the Diagnosis and Management of Chronic Heart Failure in the Adult: a report of the American College of Cardiology/American Heart Association Task Force on Practice Guidelines (Writing Committee to Update the 2001 Guidelines for the Evaluation and Management of Heart Failure): developed in collaboration with the American College of Chest Physicians and the International Society for Heart and Lung Transplantation: endorsed by the Heart Rhythm Society. Circulation. 2005;112(12):e154-e235. 16160202 10.1161/CIRCULATIONAHA.105.167586

[28] Kavalieratos D, Gelfman LP, Tycon LE, Palliative care in heart failure: rationale, evidence, and future priorities. J Am Coll Cardiol. 2017;70(15):1919-1930. doi:10.1016/j.jacc.2017.08.036 28982506 PMC5731659

[29] Mentz RJ, O’Connor CM, Granger BB, Palliative care and hospital readmissions in patients with advanced heart failure: insights from the PAL-HF trial. Am Heart J. 2018;204:202-204. doi:10.1016/j.ahj.2018.07.010 30100051 PMC6657804

[30] Rogers JG, Patel CB, Mentz RJ, Palliative care in heart failure: the PAL-HF randomized, controlled clinical trial. J Am Coll Cardiol. 2017;70(3):331-341. doi:10.1016/j.jacc.2017.05.030 28705314 PMC5664956

[31] Bekelman DB, Allen LA, McBryde CF, Effect of a collaborative care intervention vs usual care on health status of patients with chronic heart failure: the CASA randomized clinical trial. JAMA Intern Med. 2018;178(4):511-519. doi:10.1001/jamainternmed.2017.8667 29482218 PMC5876807

