# Supplementary material for: Evaluation of a Transitional Care Program After Hospitalization for Heart Failure in an Integrated Health Care System
Source: JAMA Netw Open. 2020 Dec 3;3(12):e2027410. doi: 10.1001/jamanetworkopen.2020.27410 (PMC7716192; doi:10.1001/jamanetworkopen.2020.27410)
Supplement: Supplement. — eTable. Characteristics of HF-TCP Patients Who Were Readmitted vs not Readmitted [file jamanetwopen-e2027410-s001.pdf]

Supplementary Online Content

Baecker A, Meyers M, Koyama S, et al. Evaluation of a transitional care program after hospitalization for heart failure in an integrated health care system. *JAMA Netw Open*. 2020;3(12):e2027410. doi:10.1001/jamanetworkopen.2020.27410

eTable. Characteristics of HF-TCP Patients Who Were Readmitted vs Not Readmitted

This supplementary material has been provided by the authors to give readers additional information about their work.

©2020 Baecker A et al. *JAMA Network Open*.

eTable. Characteristics of HF-TCP Patients Who Were Readmitted vs Not Readmitted

	30-day inpatient or observation stay readmission		
	No (n= 21,390)	Yes (n= 4,738)	Total (n= 26,128)
Year (row %)			
2013	4493 (80%)	1122 (20%)	5615 (21.5%)
2014	3565 (81.8%)	793 (18.2%)	4358 (16.7%)
2015	3395 (81.2%)	786 (18.8%)	4181 (16%)
2016	3483 (82.9%)	718 (17.1%)	4201 (16.1%)
2017	3400 (83.4%)	676 (16.6%)	4076 (15.6%)
2018	3054 (82.6%)	643 (17.4%)	3697 (14.1%)
Died within 30 days of discharge	305 (1.4%)	328 (6.9%)	633 (2.4%)
**Socio-demographics**			
Age	72.6 (13.6)	74.5 (12.8)	72.9 (13.5)
Gender: Female	9139 (42.7%)	2109 (44.5%)	11248 (43%)
Race/Ethnicity			
Black	3335 (15.6%)	706 (14.9%)	4041 (15.5%)
Hispanic	5534 (25.9%)	1162 (24.5%)	6696 (25.6%)
Other	1863 (8.7%)	401 (8.5%)	2264 (8.7%)
White	10658 (49.8%)	2469 (52.1%)	13127 (50.2%)
Marital Status: Partnered	11122 (52%)	2401 (50.7%)	13523 (51.8%)
Insurance Status			
Commercial/Private	5760 (26.9%)	1028 (21.7%)	6788 (26%)
Medicaid or Dual	1330 (6.2%)	306 (6.5%)	1636 (6.3%)
Medicare	14139 (66.1%)	3377 (71.3%)	17516 (67%)
Other	161 (0.8%)	27 (0.6%)	188 (0.7%)
Received medical financial assistance[a]	1871 (8.7%)	544 (11.5%)	2415 (9.2%)
Missed appointments[a]	14814 (69.3%)	3603 (76%)	18417 (70.5%)
**Clinical characteristics**			
Weighted Charlson Index	6.3 (3.2)	7.1 (3.3)	6.4 (3.2)
Cancer	4536 (21.2%)	1188 (25.1%)	5724 (21.9%)
Cerebrovascular disease	6228 (29.1%)	1671 (35.3%)	7899 (30.2%)
Chronic pulmonary disease	13047 (61%)	3093 (65.3%)	16140 (61.8%)
Dementia	1244 (5.8%)	351 (7.4%)	1595 (6.1%)
Diabetes with complications	9060 (42.4%)	2247 (47.4%)	11307 (43.3%)
Myocardial infarction	8545 (39.9%)	2115 (44.6%)	10660 (40.8%)
Hypertension - complicated	8340 (39.0%)	2306 (48.7%)	10646 (40.7%)
Renal disease	12024 (56.2%)	3042 (64.2%)	15066 (57.7%)
CKD Stage >=4	2456 (11.5%)	737 (15.6%)	3193 (12.2%)
Ejection fraction (EF) – mean (SD)	44.2 (15.9)	45.0 (16.2)	44.3 (16.0)
EF <40	8285 (38.7%)	1762 (37.2%)	10047 (38.5%)
Vasodilator[b]	5195 (62.7%)	1187 (67.4%)	6382 (63.5%)

©2020 Baecker A et al. *JAMA Network Open*.

	30-day inpatient or observation stay readmission		
	No (n= 21,390)	Yes (n= 4,738)	Total (n= 26,128)
Beta blocker[c]	5123 (61.8%)	1189 (67.5%)	6312 (62.8%)
Diuretic[d]	11189 (52.3%)	2810 (59.3%)	13999 (53.6%)
LACE readmission score			
<7	1633 (7.6%)	199 (4.2%)	1832 (7%)
7-10	8382 (39.2%)	1379 (29.1%)	9761 (37.4%)
11+	11375 (53.2%)	3160 (66.7%)	14535 (55.6%)
Laboratory acute physiology score (LAPS2)	84.1 (28.9)	90.9 (30.7)	85.3 (29.4)
Length of stay	3 (2, 5)	3 (2, 5)	3 (2, 5)
Code status: do not resuscitate	3469 (16.2%)	956 (20.2%)	4425 (16.9%)
Functional status[e]			
Missing	487 (2.3%)	108 (2.3%)	595 (2.3%)
Non-ambulatory	2637 (12.3%)	811 (17.1%)	3448 (13.2%)
Ambulates w/assistance	11780 (55.1%)	2644 (55.8%)	14424 (55.2%)
Ambulates independently	6486 (30.3%)	1175 (24.8%)	7661 (29.3%)
**Exposure to HF-TCP components**			
Home health visit or telecare	14344 (83.0%)	2931 (17.0%)	17275 (66.1%)
KP Home Health[f]	6846 (82.7%)	1436 (17.3%)	8282 (31.7%)
Non-KP Home Health[f]	4329 (82.4%)	926 (17.6%)	5255 (20.1%)
Telecare call[f]	4421 (84.8%)	793 (15.2%)	5214 (20.0%)
No 2-day home health visit or telecare	7046 (19.6%)	1807 (20.4%)	8853 (33.9%)
Care manager call	19248 (84.1%)	3631 (15.9%)	22879 (87.6%)
Provider clinic visit	16181 (85.5%)	2752 (14.5%)	18933 (72.5%)

Values are presented as mean(SD) or n(%)
[a]Collected 12 months prior to index hospitalization
[b]Vasodilator use in 12 months prior to index hospitalization for patients with EF<40% (ACE, ARB, ARNI or hydralazine-nitrate)
[c]Beta blocker use in 12 months prior to index hospitalization for patients with EF<40% (metoprolol, carvedilol, bisoprolol)
[d]Diuretic use in 12 months prior to index hospitalization for all patients (furosemide or bumetanide)
[e]Within 24 hours of hospital discharge
[f]These categories are not mutually exclusive
*Missing: age (n=41), LAPS2 (n=7)

©2020 Baecker A et al. *JAMA Network Open*.
